# Incorporation of Biomass-Based Carbon Nanoparticles into Polysulfone Ultrafiltration Membranes for Enhanced Separation and Anti-Fouling Performance

**DOI:** 10.3390/nano11092303

**Published:** 2021-09-04

**Authors:** Zhiyu Zheng, Jingwen Chen, Jiamin Wu, Min Feng, Lei Xu, Nina Yan, Hongde Xie

**Affiliations:** 1 College of Chemistry, Chemical Engineering and Materials Science, Soochow University, Suzhou 215123, China; 20194209202@stu.suda.edu.cn; 2 Key Laboratory for Protected Agricultural Engineering in the Middle and Lower Reaches of Yangtze River, Ministry of Agriculture and Rural Affairs, Institute of Agricultural Facilities and Equipment, Jiangsu Academy of Agricultural Sciences, Nanjing 210014, China; chenjingwen@jaas.ac.cn (J.C.); wujiamin012@163.com (J.W.); fengmin8156@163.com (M.F.); xulei@jaas.ac.cn (L.X.)

**Keywords:** biomass, carbon nanoparticles, hydrophilicity, anti-fouling, ultrafiltration

## Abstract

Functionalized carbon nanomaterials are considered to be an efficient modifier for ultrafiltration membranes with enhanced performance. However, most of the reported carbon nanomaterials are derived from unsustainable fossil fuels, while an extra modification is often essential before incorporating the nanomaterials in membranes, thus inevitably increasing the cost and complexity. In this work, novel functionalized biomass-based carbon nanoparticles were prepared successfully from agricultural wastes of corn stalks through simple one-step acid oxidation method. The obtained particles with the size of ~45 nm have excellent dispersibility in both aqueous and dimethyl formamide solutions with abundant oxygen-containing groups and negative potentials, which can endow the polysulfone ultrafiltration membranes with enhanced surface hydrophilicity, larger pore size, more finger-like pores, and lower surface roughness. Therefore, the separation and anti-fouling performance of membranes are improved simultaneously. Meanwhile, the addition of 0.4 wt% nanoparticles was proved to be the best condition for membrane preparation as excess modifiers may lead to particle aggregation and performance recession. It is expected that these biomass-based carbon nanoparticles are potential modifying materials for improving the separation performance and anti-fouling property of the membranes with great simplicity and renewability, which pave a new avenue for membrane modification and agricultural waste utilization.

## 1. Introduction

Carbon nanomaterials (CNMs), including carbon nanotubes, graphene, carbon nanofibers, carbon quantum dots, etc., have been extensively applied for mechanical enhancement and performance improvements in membrane separation [1,2,3]. Especially in ultrafiltration membranes, functionalized CNMs with enhanced hydrophilicity and dispersibility are expected to be an efficient modifier due to their superior water transport and sieving properties, thus greatly improving the performance and lengthening the lifetime of the membrane. For example, sulfonated graphene oxide, nitrogen-doped carbon dots, and multi-walled carbon nanotubes functionalized by isocyanate and isophthaloyl chloride groups were introduced in various kinds of ultrafiltration membranes for elevated permeability and anti-fouling properties [4,5,6]. However, these CNMs are mainly derived from unsustainable fossil fuels, which may result in resource and energy shortage. Moreover, besides the preparation process of CNMs, an extra modification of CNMs is often essential for membrane preparation, thus inevitably increasing the cost and complexity. Therefore, it is highly demanding to fabricate hydrophilic CNMs from sustainable carbon sources for membranes with extreme simplicity.

As the most abundant and renewable carbon source in nature, biomass is a potential and competitive alternative for replacing fossil-derived carbon products [7]. Research on the preparation of CNMs with carbon sources of biomass has been booming with eco-friendliness and economic efficiency. Several researchers have focused on the application of CNMs derived from purified and deep-processed biomass for membrane modification, such as cellulose [8] and glucose [9]. For example, Yu et al. prepared hydrophilic carbon nanospheres from glucose and mixed them into polyvinylidene fluoride membranes for flux and retention performance as well as anti-fouling performance improvements [9]. Similarly, Rybarczyk et al. modified membranes with chitosan-derived carbon nanodots, which also had a positive effect on the hydrophilicity of the membrane [10]. However, the preparation process of these deep-processed biomass, which contains separation and purification steps, is accompanied by time, energy, and labor consumption. CNMs derived from natural and waste biomass have also been developed over the years, while only a few have been applied in the modification of ultrafiltration membranes. Mofradi prepared mesoporous carbon microspheres from corn powder by hydrothermal process. After being modified with polydopamine (PDA), the composites of microspheres-PDA were introduced in PVDF matrix in the presence of polyethylene glycol for improving the membrane performance, which was still quite complex for membrane modification [11]. Mahat et al. prepared carbon quantum dots after carbonization-activation of oil palm biomass and then introduced them in polysulfone (PSF) membranes by soaking, thus resulting in enhanced water flux and antibacterial performance [12]. However, the dots were fabricated with complex procedures and could easily fall off of membranes without long-term lifetime. Therefore, it is still of great desire to a simple and efficient method to prepare functionalized CNMs from natural or waste biomass for membrane modification.

In this study, the biomass of corn stalks (CSs), one of the most abundant agricultural crude wastes on the earth, was utilized as the raw material for producing functionalized carbon nanoparticles with abundant surface functional groups by an acid oxidation method. Namely, the biomass-derived carbon nanoparticles (BCNPs) were prepared and functionalized in one step, which is quite simple and convenient. The carbon products were found to have a good dispersion in a wide range of solvents including water, ethanol, and *N, N*-dimethyl formamide (DMF) owing to its nanoscale particle size and strong negative potentials. Therefore, the BCNPs solution can be utilized directly for membrane preparation without irreversible agglomeration of the additives. Afterwards, PSF was utilized as the matrix of the membranes and BCNPs were added in membranes for performance improvements. The morphologies and surface hydrophilicity of the membranes were carefully characterized. The separation and anti-fouling properties of the membranes were also studied. 

## 2. Materials and Methods

### 2.1. Materials

Commercialized PSF (Udel®P-3500) was kindly supplied by Solvay (Brussels, Belgium). Ethanol and DMF with analytical grade were obtained from Sinopharm Chemical Reagent Co., Ltd (Shanghai, China). The powder of CSs was provided from local suppliers. Concentrated sulfuric acid (H_2_SO_4_, 98%) was brought from Nanjing Chemical Reagent Co., Ltd (Nanjing, China). Bovine serum albumin (BSA, 67 kDa) was obtained from Aladdin (Shanghai, China). Phosphate buffer saline (PBS) tablets were brought from MP Biomedicals LLC (Santa Ana, CA, USA). All chemicals were used without further purification. Deionized water was applied in all experiments.

### 2.2. Synthesis of BCNPs

In this study, CSs were used as carbon sources for preparing BCNPs. First, the powder was washed with deionized water three times to remove any residual dust and impurity. They were then dried in an oven at 60 °C for 24 h. After that, 0.6 g as-washed powder was added to 50 mL H_2_SO_4_ and the mixture was mildly stirred at 100 °C for 6 h. The mixture was thereafter diluted five times with deionized water and centrifuged at 5000 rpm for 10 min as it cooled down to room temperature. The centrifugation was repeated three times with deionized water until the solution became neutral. Ethanol was further applied for centrifugation to remove the residual water. After that, DMF with the volume of 35 mL was added to the precipitate and the mixture was subsequently ultrasonicated for 10 min. The mixture was continued to centrifuge at 8000 rpm for 10 min and a dark solution was thus obtained, which indicates the successful preparation and dispersion of the carbon nanomaterials in DMF. The dark DMF solution (named as BCNPs-DMF) was then removed and used for preparing the composite membrane. After vacuum drying several milliliters of the solution, the mass fraction of BCNPs in DMF solution can be calculated as 0.1 wt%.

### 2.3. Fabrication of PSF/BCNPs Composite Membranes

**Figure 1:**
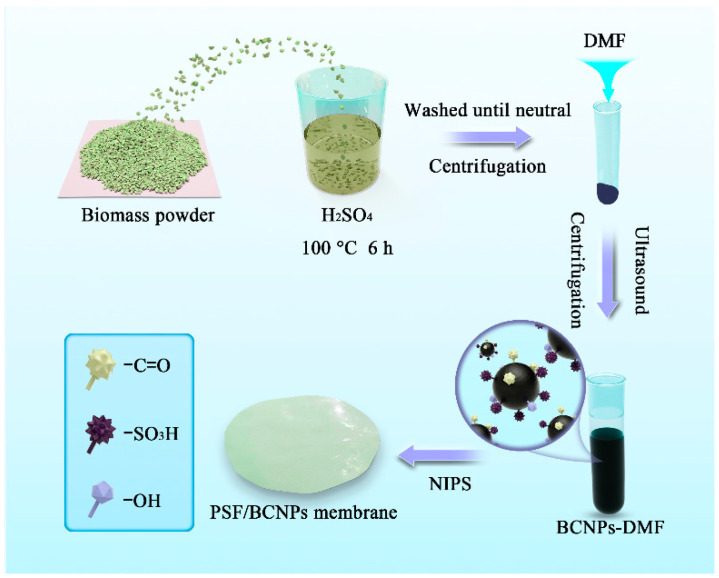
Fabrication process of PSF/BCNPs composite membranes.

The pristine PSF membrane and PSF/BCNPs composite membranes were fabricated by the nonsolvent-induced phase separation (NIPS) method [13], where deionized water was utilized as the nonsolvent. To be specific, a designated amount of PSF was dissolved in the mixture of DMF and BCNPs-DMF with various weight ratios (Table 1) at 60 °C for 12 h. The as-dissolved PSF solution was then placed in a vacuum oven for 6 h to remove any air bubbles. The film was subsequently prepared by casting the PSF solution on a clean and smooth glass with the film blade set at the height of 150 μm, followed with being coagulated in deionized water after 6 s. The as-prepared membranes were washed with deionized water and stored in water for 12 h for further characterizations. The PSF/BCNPs composite membranes were labeled as PSF-X, where X indicates the weight ratio of the BCNPs in PSF matrix (0, 0.1, 0.3, 0.4, and 0.5, respectively). For example, PSF-0.1 was prepared by 17.0 wt% PSF, 17.0 wt% BCNPs-DMF, and 66.0 wt% DMF while the total amount of BCNPs in the membrane was calculated to be 0.1 wt%. The schematic diagram of the preparation process of the membranes was shown in Figure 1.

### 2.4. Characterizations of BCNPs

To characterize the physical and chemical properties of BCNPs, the BCNPs-DMF was vacuum dried at 100 °C till the powder was obtained. Fourier transform infrared (FT-IR) spectra of the CSs and BCNPs were obtained with a Nicolet IS50 FT-IR spectrometer (Thermo Fisher Scientific, Waltham, MA, USA). The X-ray photoelectron spectroscopy (XPS, EXCALAB 250 XI, Thermo Scientific, Waltham, MA, USA) was used to analyze chemical compositions of the CSs and BCNPs. Transmission electron microscope (TEM, Tecnai G20, FEI, Hillsboro, OR, USA) was applied to characterize the morphology of the BCNPs. The average particle size and zeta potential of the nanoparticles were confirmed with the dynamic light-scattering method (DLS, Malvern Zetasizer Nano ZSE, Malvern Panalytical, Malvern, UK).

### 2.5. Characterizations of the Membranes

The surface and cross-sectional morphologies of the membranes were examined with a scanning electron microscope (SEM, SU8010, Hitachi, Tokyo, Japan) at the accelerating voltage of 5 kV. The conductivity of the samples was improved by vacuum coating with a thin layer of gold. The roughness of membranes was investigated by three-dimensional atomic force microscopy (AFM, Dimension Icon, Bruker, Madison, WI, USA) with the contact mode while the average values of surface roughness (R_a_), root roughness (R_q_), and the difference between highest peak and lowest valleys (R_z_) were obtained. The thermal stability of membranes was examined within the range of 25 °C to 800 °C by a thermal gravimetric analyzer (TGA, DTA7200, Hitachi, Tokyo, Japan) under nitrogen atmosphere with a heating rate of 10 °C min^−1^. The surface hydrophilicity of the membranes was determined with a contact angle goniometer (CA, DSA100S, Kruss, Hamburg, German). Each sample was measured for at least six different positions and the average values were recorded. The mean pore sizes (*r_m_*) of the membranes were estimated with Equation (1) as follows [14]:

(1) $$r_{m}=\sqrt{\frac{\left(2.9-1.75\varepsilon \right)\times 8\times \mu \times l\times Q}{\varepsilon \times A\times ∆P}}$$

where *μ* is the viscosity of water (1.005 × 10^−3^ Pa·s at 20 °C); *l* is the thickness of the membrane (150 μm); *Q* is the volume of water permeating PSF membrane per unit time (m^3^ s^−1^); *A* is the effective area of the membrane (3.5 × 10^−4^ m^2^); and ∆*P* is the applied pressure (0.1 MPa). *ε* is the overall porosity of the membranes which can be calculated by following Equation (2) [15].

(2) $$\varepsilon =\frac{\frac{m_{1}-m_{2}}{\rho_{w}}}{\frac{m_{1\;}-m_{2}}{\rho_{w}}+\frac{m_{2}}{\rho_{p}}}\times 100\%\;\;$$

where *m*_1_ and *m*_2_ are the weights of the wet and the dry membranes, respectively (g), and *ρ_w_* and *ρ_p_* are the densities of water (0.998 g cm^−3^ at 20 °C) and PSF, respectively.

### 2.6. Performances of the Membranes

The separation and anti-fouling properties of the membrane with the diameter of 2.2 cm were conducted with a dead-end stirred cell (UHP-25K, Advantec, Tokyo, Japan) at room temperature (20 °C) with the pressure of 0.1 MPa. A 10 min prepressing of membranes was applied in all experiments. The pure water flux (PWF, deionized water as the feed solution) was calculated based on the following Equation (3):

(3) $$J=\frac{V}{A\times t}$$

where *J* is the water (permeate) flux (L m^−2^ h^−1^), LMH); *V* is the permeate volume (L); *A* is the effective area of the membrane (3.5 × 10^−4^ m^2^); and *t* is the measurement time (h). 

The rejection properties were tested using BSA as the model. Before the test, PBS tablets were first dissolved in deionized water for preparation of the PBS solution. BSA feed solution was then prepared by dissolving BSA in PBS solution with a concentration of 0.5 g L^−1^. Concentrations of feed and permeate solutions were determined by UV-vis spectrophotometer (NanoDrop ONE^C^, Thermo, Waltham, MA, USA) under the absorption wavelength of 280 nm. The rejection rate (*R*) was obtained by Equation (4):

(4) $$R=\left(1-\frac{C_{P}}{C_{f}}\right)\times 100\%$$

where *C_p_* and *C_f_* represent BSA concentrations of permeate and feed solutions, respectively (g L^−1^).

The anti-fouling properties of these membranes were investigated by testing the flux recovery ratio (*FRR*), total flux decreasing ratio (*DR_t_*), reversible flux decreasing ratio (*DR_r_*), and irreversible flux decreasing ratio (*DR_ir_*), respectively. The original PWF were measured every 10 min and the total PWF for 1 h was recorded as *J*_1_. BSA solution was then used as the feed and the flux was also measured according to Equation (4) every 10 min with the total flux for 1 h recorded as *J*_2_. After that, the contaminated membrane and the manipulated cell were thoroughly cleaned with deionized water. Afterwards, deionized water was reused as the feed and the PWF were recorded again for 1 h as *J*_3_. The obtained time-dependent values were normalized to clearly show the changes in water fluxes. The antifouling performance of the prepared membrane was evaluated using the following Equations (5)–(8) [16].

(5) $$FRR=\frac{J_{3}}{J_{1}}\times 100\%$$

(6) $$DR_{t}\left(\%\right)=\left(1-\frac{J_{2}}{J_{1}}\;\right)\times 100\%$$

(7) $$DR_{r}\left(\%\right)=\left(\frac{J_{3\;}-J_{2}}{J_{1}}\right)\times 100\%$$

(8) $$DR_{ir}\left(\%\right)=\left(\frac{J_{1}-J_{3}}{J_{1}}\right)\times 100\%$$

## 3. Results and Discussion

### 3.1. Characterizations of BCNPs

To prepare functionalized BCNPs, the as-washed powder of CSs was carbonized in H_2_SO_4_ and separated from the solution by centrifugation. It was found that the upper solution became darker with the centrifugation times which may mainly be ascribed to the recession of the acidity of the solution. The acid environment is helpful for centrifugation as it can reduce the absolute potential of carbon nanomaterials [17]. Therefore, as the acid environment weakens, the solubility of BCNPs increases with the increased absolute potential, thus leading to the darkness of the solution. 

**Figure 2:**
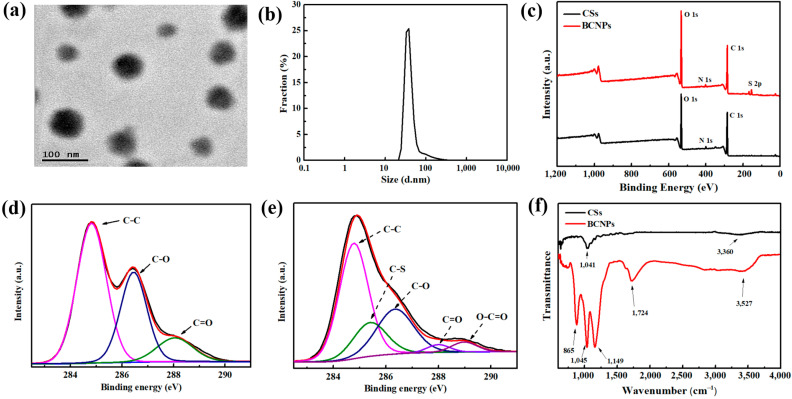
(**a**) The TEM image and (**b**) size distribution of BCNPs, (**c**) the XPS spectra of CSs and BCNPs, the XPS C1s spectra of (**d**) CSs and (**e**) BCNPs, (**f**) the FT-IR spectra of CSs and BCNPs, respectively.

The morphology of the BCNPs is shown in Figure 2a. Some round particles with the mean size of ~45 nm were observed, which was also confirmed by the DLS result (Figure 2b). This indicates BCNPs derived from carbonization of biomass in H_2_SO_4_ are much smaller than those prepared by calcination which are detailed in our previous work [18]. To compare the compositions (C, S, O, N) between CSs and BCNPs, XPS was employed and the results were given in Figure 2c and Table 2. Compared with the spectrum of original biomass, an obvious increase of O content and the appearance of a new element of S were observed with BCNPs. Meanwhile, the C 1s spectra of CSs and BCNPs were also given in Figure 2d,e. It is noted that two peaks at 285.3 eV and 289 eV were only observed with the C 1s spectrum of BCNPs, which represents the formation of C–S and O–C=O bonds [19]. The FT-IR spectra of CSs and BCNPs were depicted in Figure 2f. It is noted that new peaks centering at 865 cm^−1^ and 1149 cm^−1^ should correspond to the –SO_3_H stretching vibration with BCNPs samples. Meanwhile, the peaks centering around 1724 cm^−1^ and 3527 cm^−1^ represent the –C=O and –OH stretching vibrations, respectively. The XPS and FT-IR results both indicate the existence of O- and S-containing functional groups after carbonization, which was expected to render BCNPs with hydrophilicity and electronegativity. This indicates that the functionalized BCNPs are successfully prepared with the one-step acid treatment. The potential of BCNPs was measured as −42.9 mV, therefore the BCNPs can be quite stable after being dispersed in aqueous or DMF solutions with repulsive interactions [20]. 

### 3.2. Characterizations of PSF/BCNPs Composite Membranes

**Figure 3:**
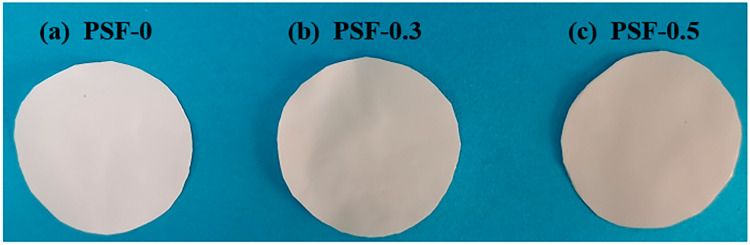
The macroscopic images of (**a**) PSF-0, (**b**) PSF-0.3, and (**c**) PSF-0.5 composite membranes.

**Figure 4:**
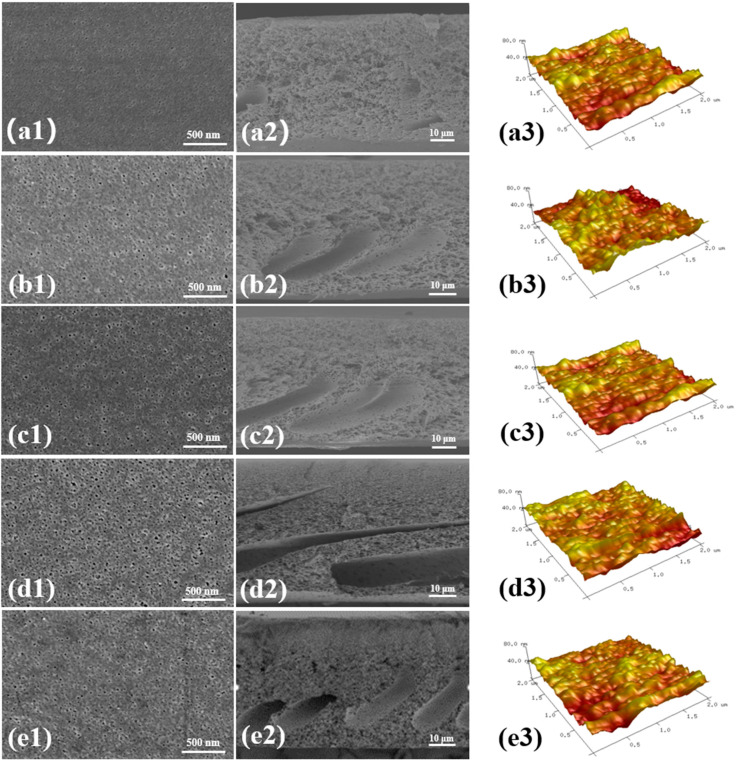
(**a1**–**e1**) The surface, (**a2**–**e2**) cross-section, and (**a3**–**e3**) three-dimensional AFM images of PSF/BCNPs composite membranes.

After dispersing BCNPs in DMF, the PSF/BCNPs solution was further prepared by dissolving a designated amount of PSF in the mixture of BCNPs-DMF and DMF. The pristine PSF membrane and PSF/BCNPs composite membranes were fabricated by these solutions following the NIPS method. The macroscopic morphologies of the membranes are shown in Figure 3. It is observed that the color of the membrane gradually darkens with the increase of BCNPs content. The microscopic surface morphologies of the membranes were characterized by SEM and the images were given in Figure 4(a1–e1). Relatively smooth surface with some small round pores can be observed at the surface of all the membranes. However, with the introduction of BCNPs, the pores seem to be more obvious and larger, which was confirmed by the pore size values listed in Table 3. The mean pore size of the modified membranes measured by Nanomeasurer or calculated from Equation (1) indicated much larger pores in PSF/BCNPs composite membranes at the same time. This should be ascribed to the addition of hydrophilic BCNPs which accelerates the solvent exchange rate, thus leading to the formation of larger pores. Meanwhile, we also noted that PSF-0.4 had the largest pore size of any other membrane. As the content of BCNPs increased to 0.5 wt%, the mean pore size slightly declined. It is speculated that excessive addition will lead to agglomeration of BCNPs, thus hindering the solvent exchange rate and decreasing the pore size. We also examined the cross-section morphologies of the membranes. As shown in Figure 4(a2–e2), all of the membranes were highly porous with quite uniform thickness. Meanwhile, there was no obvious difference in thickness among the membranes with varied BCNPs contents as listed in Table 3. Asymmetric structures featured with a dense top layer and a highly porous sublayer were observed in all membranes. Sponge-like and finger-like pores can also be observed in all membranes. However, we note that more finger-like pores were formed with the addition of BCNPs. During the solvent exchange process, the diffusion of DMF accelerates in water with the addition of hydrophilic BCNPs, which result in the formation of more finger-like pores [21,22]. Therefore, the modified membranes have a less dense top layer and more highly porous sublayer. Meanwhile, the porosities of the membranes were slightly increased with BCNPs contents which were calculated by Equation (2) and listed in Table 3.

AFM images of the membranes with the 2 μm × 2 μm size, which are given in Figure 4(a3–e3), were characterized for quantitatively determining the surface roughness. R_a_, R_q_, and R_z_ were listed in Table 3. We note that the roughness of the modified films was decreased compared with those of the pristine membrane and PSF-0.4 has the lowest value. For example, the R_a_, R_q_, and R_z_ of PSF-0 were 7.3 nm, 5.6 nm, and 53.4 nm. With the addition of only 0.1 wt% BCNPs, these three parameters decreased to 7.0 nm, 5.5 nm, and 51.0 nm, respectively. As for PSF-0.4, the roughness was further decreased to 5.6 nm, 4.5 nm, and 34.8 nm. Further increasing the BCNPs content leads to an addition of the roughness to 6.2 nm, 5.0 nm, and 39.1 nm, respectively. The O-containing groups have great effects on the surface properties of the PSF/BCNPs membranes with the creation of electrostatic forces among polymer chains, chains wrinkling, bending and compactness, etc. Therefore, BCNPs are expected to migrate and distribute uniformly on the membrane surface during the membrane preparation process, thus leading to the decrease of surface roughness. Excess nanoparticles with the content of 0.5 wt% may agglomerate during the process, and the uneven distribution of BCNPs was expected on membrane surface, which result in the increase of surface roughness [23]. 

**Figure 5:**
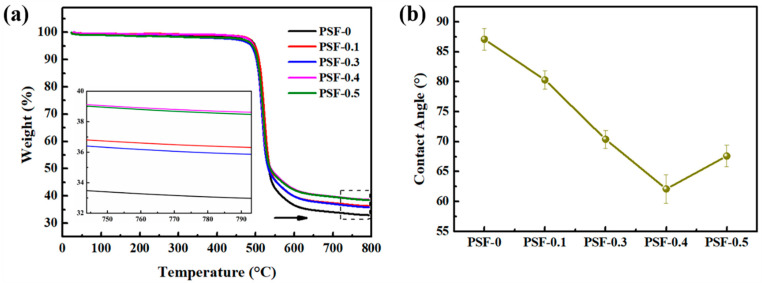
(**a**) The TGA and (**b**) CA curves of the PSF/BCNPs composite membranes.

The thermal stability of the membranes was characterized by TGA, and the curves were given in Figure 5a. The pristine membrane decomposed at the temperature of 500 °C in the nitrogen atmosphere, which is coincident with other research [24]. The decomposition temperature of the other composite membranes seems have no obvious change with the introduction of BCNPs. This indicates the PSF-based membranes have high temperature resistance and the thermal degradation mechanism nearly has no change with BCNPs. As the temperature further increased from ~520 °C to 800 °C, the mass ratio of the samples was decreased continuously and steadily. Noticeably, the residual mass of the membrane was gradually increased with the raised proportion of BCNPs, for example, the residual mass percentage increased from 32.9% of PSF-0 to 35.8% with PSF-0.1. It further increased to 36.3%, 38.5%, and 38.4% for PSF-0.3, PSF-0.4, and PSF-0.5, respectively. Considering the fine distinction of BCNPs contents among the membranes, the difference of the residual mass percentage should result from the interactions between PSF and BCNPs. BCNPs with a great number of O-containing functional groups are able to be hydrogen bonded with the –SO_2_– and –O– of PSF molecular chains, which restricts the mobility of PSF molecular chains in the pyrolysis process and eventually slightly improves the residual weight of composite membranes [9,24]. 

Considering that the separation and anti-fouling performance of the membranes were greatly influenced by its surface properties, the surface hydrophilicity of membranes were characterized by measuring CA and the results were depicted in Figure 5b. For PSF-0, the CA is measured to be 87.1°, which is nearly same as the value reported elsewhere [25]. With the addition of BCNPs, the CA decreased from 87.1° (PSF-0) to 62.1° (PSF-0.4), indicating that BCNPs have a positive effect on the membrane hydrophilicity. However, the CA was increased as the BCNPs proportion raised from 0.4 wt% to 0.5 wt%, which may probably be owing to the agglomeration of the BCNPs and the blockage of membrane pores by the excessive amounts of BCNPs [26].

### 3.3. Performance of PSF/BCNPs Composite Membranes

**Figure 6:**
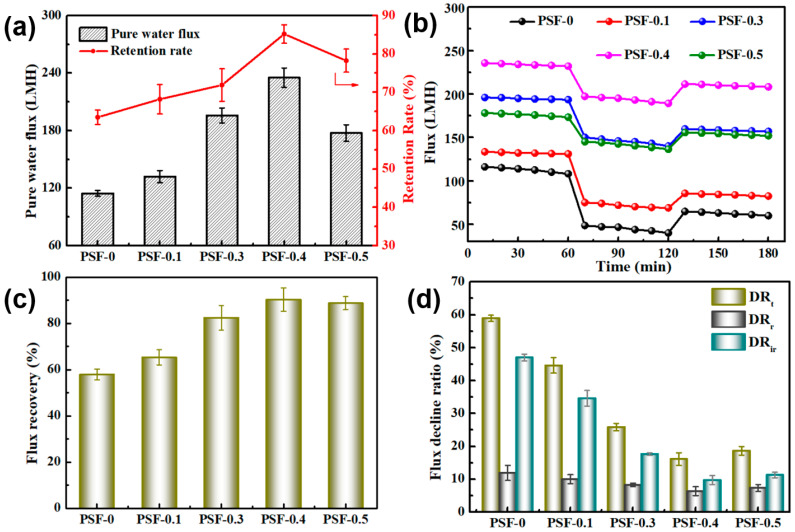
(**a**) PWF and BSA rejection, (**b**) time-dependent flux, (**c**) *FRR*, and (**d**) *DR* of the PSF/BCNPs composite membranes.

The separation performance of membranes was first characterized by testing the PWF and BSA rejection rates. The results were calculated by Equations (3) and (4) with the value given in Figure 6a. It is observed that all PSF/BCNPs composite membranes exhibit higher PWF than PSF-0 while PSF-0.4 has the highest flux value. The flux of PSF-0.4 with the value of 235.2 LMH is more than twice the flux of PSF-0 which is only 114.6 LMH. This tremendous increase of PWF should benefit from the synergistic increase of the surface hydrophilicity as well as pore size of the membranes. The decreased surface roughness and increased number of finger-like pores are also believed to have a positive effect on PWF as they can decrease mass transfer resistance and accelerating transfer rate of water molecules [9]. However, the surface hydrophilicity and pore size of the membranes were both decreased while surface roughness of them was increased as the BCNPs content increased from 0.4 wt% to 0.5 wt%, which results in the decrease of PWF of the membrane.

Even though the surface pore size of the membranes was increased with the addition of BCNPs, the rejection rate of BSA was surprisingly enhanced as well, as observed in Figure 6a. The rejection rate of BSA was increased from 63.5% for PSF-0 to 68.2%, 71.9%, 85.2%, and 78.3% with the BCNPs content increased to 0.1 wt%, 0.3 wt%, 0.4 wt%, and 0.5 wt%, respectively. This indicated that the introduction of BCNPs could simultaneously upgrade the permeability and retention of the membranes. Notably, PSF-0.4 not only have the highest permeability, but also have the highest BSA retention rate. It is believed that BSA molecules are prone to absorb on the pristine PSF membranes due to surface hydrophobicity. However, the surface hydrophilicity of the membranes increased with the existence of BCNPs, thus forming a thin layer of water on the membrane surface by hydrogen bond [9]. The thin layer of water is expected to hinder BSA from absorbing on and going across the membranes which results in the enhancement of BSA rejection. Compared with that of PSF-0, the BSA rejection rate of the PSF-0.4 is increased by 21.7%, which is superior to many other CNMs-based modifiers as listed in Table 4.

The fouling of PSF membranes which results from inherent surface hydrophobicity is a nonnegligible and complex problem, which will lead to the decline of membrane performance and service lifetime [25]. Therefore, it is very important to enhance the hydrophilicity of the PSF membrane. The anti-fouling properties of BCNPs-incorporated membranes are expected to be greatly enhanced by introducing BCNPs, as the surface hydrophilicity was proved with an improvement. The time-dependent flux of the prepared membranes before (0–60 min), during (60–120 min), and after (120–180 min) BSA filtration is shown in Figure 6b. All the membranes have a declined flux with the feed solution replaced by BSA solution, resulting from the absorbed BSA molecules on the surface and pore walls of the membranes. However, all of the modified membranes exhibited higher flux compared to that of the bare PSF during the whole process. Meanwhile, the *FRR*, *DR_t_*, *DR_r_*, and *DR_ir_* were calculated by Equations (5)–(8) to further characterize the anti-fouling properties of the prepared membranes and the results are shown in Figure 6c,d. *DR_r_* reflects the amount of the absorbed BSA on the membrane surface and pores wall that can be easily washed away by deionized water. Correspondingly, the foulant which cannot be removed by washing leads to *DR_ir_*. *DR_ir_* is the direct reason for membrane performance decline. It is observed that *FRR* of the PSF/BCNPs composite membranes were all higher and *DR_ir_* were all smaller than those of pristine PSF membrane, which confirms that the BCNPs could greatly enhance the anti-fouling properties of the PSF membranes. Meanwhile, the PSF-0.4 shows the highest *FRR* of 90.3% and lowest *DR_ir_* of 9.7%, indicating that the addition of 0.4 wt% BCNPs is the best condition for alleviating membrane fouling. It is noted that the *FRR* of the PSF-0.4 is also superior to the vast majority of relevant studies, as shown in Table 4. The enhanced permeability and anti-fouling property of PSF/BCNPs composite membranes are both resulting from the increased membrane hydrophilicity as the incorporated BCNPs possessed rich accessible hydroxyl, sulfonic, and carbonyl groups on the particle surface. These superficial O-containing groups can form a water layer on the membrane surface which could accelerate water molecules to pass through the membrane and reduce the interactions between BSA molecules and membrane surface. Therefore, the water layer is able to hinder BSA’s adherence on the surface and pore walls of the membrane, thus enhancing the permeability, rejection rate, and anti-fouling property of the membranes simultaneously. Meanwhile, the decrease of the membrane surface roughness also plays a positive role in membrane anti-fouling properties [9] as BSA molecules are less likely to be deposited on smoother surface of the membrane [27].

**Table 4:** Performance comparison of the membranes modified with different CNMs.

Modified Materials	% Increase in BSA Retention	FRR	References
Biomass-based carbon nanoparticles	21.7	90.3%	This work
Guanidyl-functionalized graphene	0.5	82.4%	[28]
Glucose-based carbon nanospheres	~9.0	~83.0%	[9]
Sulfonated graphene oxide	nearly unchanged	–	[6]
Oxidized carbon nanotubes	21.7	72.8%	[2]
Graphene oxide	17.2	85.1%	[2]
Oxidized carbon nanotubes and graphene oxide	14.2	80.4%	[2]
Graphene oxide	~2.0	69.0%	[29]
Graphene oxide-polyethylene glycol	~−2	78.0%	[29]
Cysteine-functionalized graphene oxide	~3.0	92.1%	[19]
PDA-functionalized GO	nearly unchanged	86.9%	[30]

## 4. Conclusions

To conclude, novel functionalized BCNPs derived from CSs have been prepared by one-step acid treatment with the size of ~45 nm. It was proved that they are rich in O-containing groups with strong negative potentials and have excellent dispersibility in both aqueous and DMF solutions. The introduction of these BCNPs is expected to endow the PSF ultrafiltration membranes with enhanced surface hydrophilicity, large pore size, more finger-like pores, and smooth surface roughness, thus leading to improved permeability, BSA rejection rate, and anti-fouling properties simultaneously. Meanwhile, the addition of 0.4 wt% BCNPs was proved to be the best condition for membrane preparation, as excess BCNPs may lead to particle aggregation. The BCNPs-based membrane modification is of great simplicity, that not only paves a new avenue for excess agricultural waste treatment, but also provides a tremendous material for performance improvement in membrane technology. 

## Figures and Tables

**Table 1:** Preparation conditions for PSF/BCNPs composite membranes.

	PSF (wt%)	BCNPs-DMF (wt%)	DMF (wt%)	Total (wt%)
PSF-0	17.0	0.0	83.0	100.0
PSF-0.1	17.0	17.0	66.0	100.0
PSF-0.3	17.0	51.0	32.0	100.0
PSF-0.4	17.0	68.0	15.0	100.0
PSF-0.5	17.0	83.0	0.0	100.0

**Table 2:** XPS results and zeta potentials of CSs and BCNPs.

	Atomic Ratio (wt%)				Zeta Potential (mV)
	C	O	N	S	
CSs	64.0	33.7	2.3	0.0	/
BCNPs	52.1	41.4	2.0	4.5	−42.9

**Table 3:** Characteristic parameters of PSF/BCNPs composite membranes.

Membrane	Thickness (μm)	Porosity (%)	Mean Pore Size (nm)		Surface Roughness Parameters (nm)		
			a ^1^	b ^2^	R_a_	R_q_	R_z_
PSF-0	48.3	70.3	30.1	28.2	7.3	5.6	53.4
PSF-0.1	48.6	72.0	31.7	32.5	7.0	5.5	51.0
PSF-0.3	48.8	72.7	38.2	42.3	6.4	5.0	46.9
PSF-0.4	49.2	73.7	41.4	48.3	5.6	4.5	34.8
PSF-0.5	49.0	72.7	36.4	46.2	6.2	5.0	39.1

^1^ a is calculated by Equation (1); ^2^ b is calculated by Nanomeasurer.

## Data Availability

The data presented in this study are available upon request from the corresponding author.
